# Supplementary material for: Patterns of Intron Gain and Loss in Fungi
Source: PLoS Biol. 2004 Nov 30;2(12):e422. doi: 10.1371/journal.pbio.0020422 (PMC532390; doi:10.1371/journal.pbio.0020422)
Supplement: Table S1 — Also available at http://genes.mit.edu/NielsenEtAl/. (4.3 MB ZIP). [file pbio.0020422.st001.zip › NielsenEtAl/html/435.html]

AN6251.1.NCU01804.1.MG06680.1.FG10876.1


```
 CLUSTAL W (1.82) Multiple Sequence Alignments - Introns Inserted


Sequence 1: NCU01804.1	164 aa
Sequence 2: MG06680.1	168 aa
Sequence 3: FG10876.1	164 aa
Sequence 4: AN6251.1	159 aa
Alignment Length: 171 aa
Number Identitical Residues: 77 aa
Alignment Score (without introns) 3787


MG06680.1 	MAASGSSN-SSTRTMQSRTGRSKQR1YNSKGERLVAGVVALSEDKEYVLLTQSTRRKGWV
NCU01804.1	MAASNTS--SGNRSMESRTGRTKQR1YNTKGERLVAGVVPLSADKYYVMLIQSTRRKGWV
FG10876.1 	MSSSASGDSGGGRSMESRVGRSKQR1YNTKGERLVAGIVPLTSDQNYVLLIQSTRRKGWV
AN6251.1  	MVD-------QVRSMESRVGRKNQR1YGSKGERLVAGVVPLSKDKSLVMMIQSAGRGGWV
          	*           *:*:**.**.:** *.:********:*.*: *:  *:: **: * ***

MG06680.1 	LPKGGWETDE-ECTEAAEREAWEEAGIVVTIDYDLGQIEETRVKTSSKSAKSGKREKAIY
NCU01804.1	LPKGGWELDE-ECHEAAAREAWEEAGIVVQINYDLGDIQDTR---PPKKNPLKEKERSLY
FG10876.1 	LPKGGWESDE-TCQEAAEREAWEEAGITVQISYDLGDIDEKR---APKSSS---KDRSRY
AN6251.1  	LPKGGWETDEASAQQAACREAWEEAGVICTVLRDLGTISDMR----PSTMLTSNSPRASY
          	******* **: . :** ********:   :  *** *.: *    ...    .  :: *

MG06680.1 	HFYEATVTSEEQDWPEKDKRQRKWMTFVDAWESLKDRPELQEALNRSTMKR-
NCU01804.1	RFFEATVTSEEPEWPEKDKRERKWYTYAEATELLKERPELQAALDLSTIKR-
FG10876.1 	HFFEGTVTGEFDEWPESHKRERQWFTFTQAWEALSTRPELQEALQRSTVKRQ
AN6251.1  	QFFEVTVDREEDRWPEMHKRRRQWVTYTQAAAALASRPELLEALNRSSMKR-
          	:*:* **  *   *** .**.*:* *:.:*   *  ****  **: *::**
```
